# Effect of zinc oxide nanoparticle supplementation on parasite infection and rumen environment of grazing lambs

**DOI:** 10.3389/fvets.2025.1684585

**Published:** 2025-10-30

**Authors:** Matej Leško, Alexandra Bombárová, Daniel Petrič, Dominika Batťányi, Michaela Komáromyová, Alžbeta Königová, Michal Babják, Ľuboš Halada, Stanislav David, Anna Łukomska, Piotr Pawlak, Pola Sidoruk, Adam Cieslak, Klaudia Čobanová, Zora Váradyová, Marián Várady

**Affiliations:** ^1^Institute of Animal Physiology of Centre of Biosciences of Slovak Academy of Sciences, Košice, Slovakia; ^2^University of Veterinary Medicine and Pharmacy in Košice, Košice, Slovakia; ^3^Institute of Parasitology of Slovak Academy of Sciences, Košice, Slovakia; ^4^Institute of Landscape Ecology of Slovak Academy of Sciences, Bratislava, Slovakia; ^5^Department of Preclinical Sciences and Infectious Diseases, Poznan University of Life Sciences, Poznan, Poland; ^6^Department of Genetics and Animal Breeding, Poznań University of Life Sciences, Poznań, Poland; ^7^Department of Animal Nutrition, Poznań University of Life Sciences, Poznań, Poland

**Keywords:** parasitological status, phytosociological relevés, zinc, rumen, histology

## Abstract

This study investigated the effect of zinc nanoparticles (ZnO-NPs) on the growth parameters, parasitological status, ruminal fermentation, and histopathology of lambs that were experimentally infected with Haemonchus contortus larvae. The infected lambs were divided into two groups (*n* = 10/group) and grazed on pasture while being fed one of two diets: a control diet consisting of 350 g/d of a dietary concentrate (CONTROL), and a diet consisting of 350 g/d of concentrate enriched with ZnO-NPs (ZINC). Pasture aboveground plant coverage and plant taxa from phytosociological relevés were used as descriptors to investigate differences in vegetation based on plant medicinal properties and the nutritional value. Communities dominated by plants with medicinal properties were mainly found in the CONTROL group's pasture, while the pasture of the ZINC group contained most plants with outstanding nutritional value. The number of eggs per gram of feces was quantified on days D14, D20, D28, D42, D56, D70, D84, D98, and D107 post-infection. There was a significant decrease in egg shedding from D42 onwards in the ZINC group, and from D56 and D70 onwards in the CONTROL group. The ruminal concentration of ammonia nitrogen (*p* = 0.018), *n*-butyrate (*p* = 0.025), n-valerate (*p* = 0.002), total protozoa count (*p* < 0.001), and the enzymatic activities of α-amylase (*p* < 0.001) and xylanase (*p* = 0.006) were significantly higher in the ZINC group than in the CONTROL group. The molar proportion of acetate was lower (*p* = 0.011) in the ZINC group than in the CONTROL group. Morphological observations of the rumen indicated that the homogeneity of the ruminal papillae was slightly impaired, the lamina propria was inflamed, or lymphocytes had infiltrated. In conclusion, the dynamics of gastrointestinal nematode infection were significantly reduced, probably due to the medicinal and nutritional properties of the pasture plants. This effect was also enhanced by the supplementation with ZnO nanoparticles, which possess strong anthelmintic potential.

## 1 Introduction

Helminth infections in grazing ruminants encompass a wide range of parasitic genera and species, which can also intensify emissions of greenhouse gases by influencing factors such as feed intake, composition, and the ruminal microbiome (1, 2). The development of new preventive and therapeutic strategies and alternative methods for controlling gastrointestinal nematodes (GINs) has intensified in recent decades, because anthelmintic therapy is considered unsustainable and non-ecological, primarily due to the accumulation of drug residues in animal products and the development of resistance to multiple anthelmintics (3, 4). Much research has centered on the use of herbs containing bioactive compounds in ruminants infected with GINs for their nutritional effects and anthelmintic properties (5, 6).

Our previous studies (6–9) have described the diversity and synergy of the bioactive compounds of several medicinal herbs and their various combinations, which contribute to some level of pharmacological efficacy. Most of these studies concluded that supplementing the diet with a mixture of dried medicinal herbs and adding some trace elements such as zinc or selenium could slow the progression of infection and increase resistance in lambs (10, 11). We more recently investigated the effect of grazing on plant species-rich semi-natural grassland enriched with experimentally sown chicory on the parasitological status, growth parameters, larval contamination of pastures, ruminal microbiota, parameters of serum antioxidants, and histology of lambs infected with *Haemonchus contortus* (12). Our results indicated that the presence of plants containing bioactive compounds and the enrichment of pastures with chicory could greatly reduce the parasitic burden in lambs and the contamination of pastures with infective nematode larvae.

These alternative anthelmintic approaches, however, have various disadvantages, including low efficacy and a lack of target selectivity, which can lead to inconsistent outcomes. Ongoing development and innovation in nanomedicine have nevertheless led to the investigation of using nanoparticles (NPs) to deliver polyphenols, antiparasitic drugs, and organic and inorganic trace elements. The increased biological activity of NPs is due to their subcellular size, enabling them to penetrate cells and enhance bioavailability, sustained release, and intracellular penetrability (13). A recent study summarized information from 18 articles on the anthelmintic effects of NPs (e.g., Ag-NPs, ZnO-NPs, and chitosan-NPs) against *H. contortus*. Thirteen of these studies reported *in vitro* efficacy, nine reported *in vivo* efficacy, and four reported both (14). Only the *in vitro* efficacy of most of the inorganic NPs (e.g., silver, gold, zinc oxide, iron oxide, and nickel oxide) against gastrointestinal helminths, however, has been studied (15, 16). NPs derived from natural substances have negligible side effects on animals (17). The majority of studies support the beneficial effects of ZnO-NPs on animal health (18, 19). These NPs are used as additives in animal feed due to their antimicrobial and immunomodulatory properties, which alter the ability of ruminal epithelial cells to transport short-chain fatty acids and thereby affect fermentation (20, 21). Several studies using various biological models and biomarkers, however, have also reported ZnO-NP toxicity, including cell death, increased oxidative stress, DNA damage, apoptosis, and an induced inflammatory response (22–24). The anthelmintic effect of ZnO-NPs is, nevertheless, their ability to induce oxidative/nitrosative stress and damage the DNA of parasites (25–27).

Research on zinc NPs is dominated by *in vitro* studies, but more *in vivo* research is needed to improve our understanding of the anthelmintic effects of ZnO-NPs in animals. We hypothesized that ZnO-NPs could reduce the population of *H. contortus* and alter the ruminal environment in infected lambs. Our goal was to investigate the effects of ZnO-NP supplementation on the parasitological status, ruminal microbiota, fermentation, and histology of lambs infected with *H. contortus* grazing on a semi-natural grassland. Our previous results (12) indicated that grassland containing plants with bioactive compounds could significantly reduce parasitic burdens in lambs and the contamination of pastures with infective nematode larvae, so we also assessed the pasture vegetation using phytosociological relevés.

## 2 Materials and methods

### 2.1 Pastures

The experiment was conducted on pastures on a private farm (PETLAMB) in Petrovce, district of Prešov, Slovakia. Two plots with areas of 0.283 ha of grassland with mixed plant species composition were selected, fenced with electric fencing, and labeled as a control plot and a plot for lambs supplemented with ZnO-NPs. The plots were equipped with an automatic water trough and a shelter. The experiment was conducted between May and September 2024.

### 2.2 Evaluation of vegetation

The vegetation on pastures was recorded on June 19, 2024, using the method of phytosociological relevés (28). The size of each relevé was 25 m^2^ (5 × 5 m). Two permanent plots were established on each pasture, where the vegetation was assessed. All plant taxa on the permanent plots were first identified, and the aboveground coverage of individual taxa was then estimated using an ordinal scale (29), a range of categories of abundance. Species growing outside of permanent plots were recorded as additional information; they were not included in quantitative analyses. The scientific names of the plant species were used according to the Slovak Vegetation Database (30). A matrix of medicinal properties and nutritional values of plant species was compiled and subjected to multivariate principal component analysis (PCA). Plant species were classified based on their medicinal properties, Raunkiær’s system of life forms, synanthropy, toxicity of plant organs, feed value, and the melliferousness of their nectar, honeydew, and pollen (31). Phytosociological records on pastures with an indication of selected medicinal properties and nutritional values are presented in Table 1.

**Table 1:** Vegetation records (number of species and percentage of cover represented by species with defined characteristics).

Pasture of a group of lambs	CONTROL plot	ZINC plot
Relevé area (m^2^)	25.0	25.0
Altitude (m)	346	351
Aspect (°)	90	90
Slope (°)	17	5
Herb coverage (%)	70	70
Number of species in relevée	59	49

	Characteristic	*N*	Ab	*N*	Ab
Medicinal property	1 homeopathy/folk medicine	16	1–4	16	2–5
	2 medicinal products	3	2–6	3	4–6
	3 pharmacopeia	11	1–5	5	2–4
Nutritional value	5 excellent	5	2–4	5	2–5
	4 very good	7	2–5	6	2–5
	3 good	3	2–5	5	2–6
	2 low to medium	13	1–5	11	1–4
	1 inferior	17	2–6	15	2–6
	–1 unsuitable	2	1–4	1	2
	–2 harmful	1	4	0	0
	–3 very harmful	2	2	1	4

CONTROL plot, a plot consisting exclusively of grassland with grazing lambs fed a diet without ZnO-NPs; ZINC plot, a plot consisting exclusively of grassland, with grazing lambs fed a diet enriched with ZnO-NPs; N, number of species; Ab, abundance, range (lowest and highest) of categories of abundance: 1, rare 1–2 individuals; 2, more individuals coverage less that 1%; 3, coverage 1–5%, low number of individuals; 4, coverage 1–5%, many individuals; 5, coverage 5–12.5%; 6, coverage 12.5–25%; 7, coverage 25–50%; 8, coverage 50–75%; 9, coverage 75–100%.

### 2.3 Animals, diets, and experimental design

**Figure 1:**
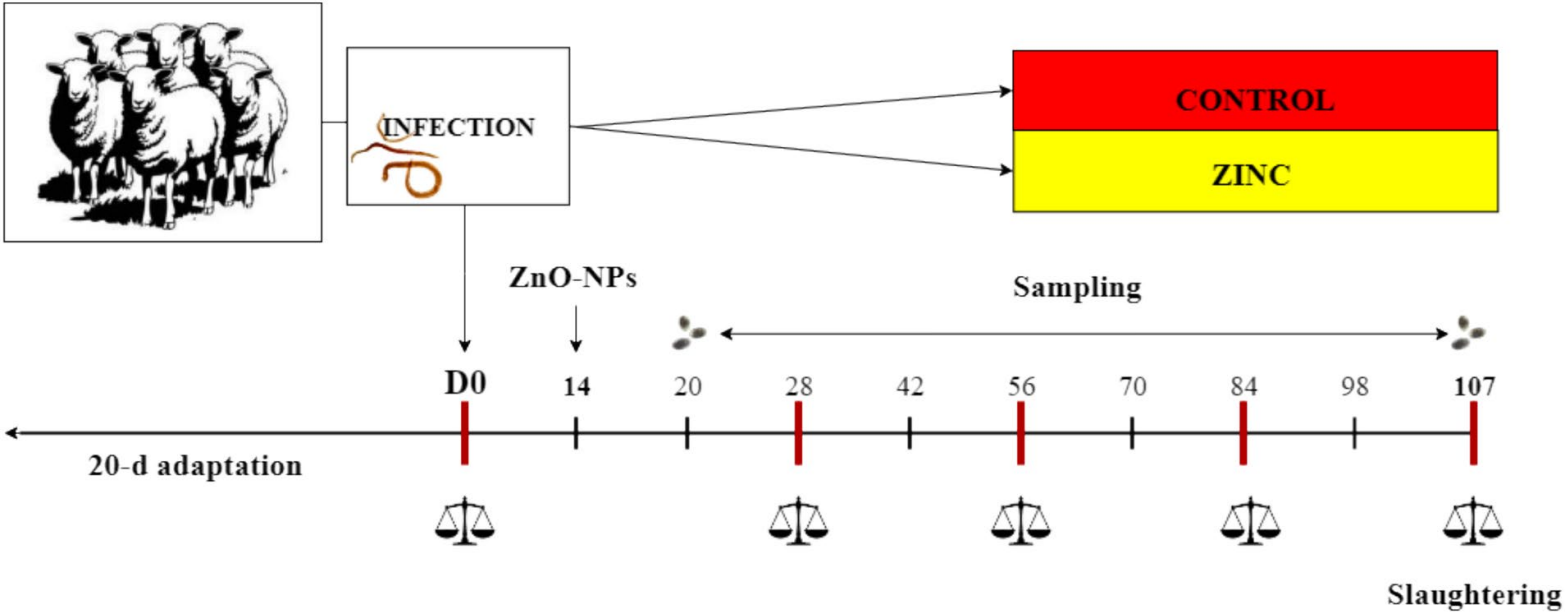
Overview of the experimental design.

Twenty male and female (1:1) Tsigai breed lambs, aged 3–4 months, purchased from a sheep farm (PD Ružín–farm Ružín, Kysak, Slovakia), were housed at the private farm. The number of animals used in the experiment was assigned according to the VICH GL13 guidelines proposed by the European Medicines Agency. The lambs were dewormed with the recommended dose of albendazole (Albendavet 1.9% susp. a.u.v; DIVASA-FARMAVIC S. A., Barcelona, Spain) during a 20-d adaptation period before the start of the experiment and subsequently tested for parasite egg production after 14 days with negative results. All parasite-free lambs were orally infected with approximately 5,000 third-stage larvae of the MHCo1 strain of *H. contortus* susceptible to anthelmintics (10). The day of infection was considered day 0 (D0) of the experiment. All lambs with body weights of 16.1 ± 2.63 kg (mean ± standard deviation) were grazed on pasture and fed one of two diets (*n* = 10/diet): (a) a diet consisting of 350 g/d of a dietary concentrate (CONTROL), and (b) a diet consisting of 350 g/d concentrate enriched with ZnO-NPs (ZINC). The concentrate contained ground barley (563 g/kg), wheat bran (282 g/kg), soybean meal (141 g/kg), and limestone (14 g/kg). The ZnO-NPs were a commercial product administered to the lambs in the ZINC group at a dose of 120 mg Zn/kg of concentrate (SkySpring Nanomaterials, Inc., Houston, USA). Aliquots of the ZnO-NPs were mixed directly with the concentrate for each meal to provide a diet with additional zinc. The ZINC group started receiving the zinc diet on D14. The lambs were weighed on D0, D28, D56, D84, and D107. The experimental period was 107 d, and the lambs were euthanized following the rules of the European Commission (32). All lambs were euthanized using an overdose of 140 mg/kg pentobarbital (Dolethal; Vetoquinol, Towcester, UK, Ltd.) at the end of the experiment at the abattoir of the Centre of Biosciences of SAS, Institute of Animal Physiology, Košice, Slovakia, No. SK U 03023. The pentobarbital overdose had a negligible effect on the estimated parameters in the lambs. The rumen and abomasum were collected from each lamb immediately after slaughter in the abattoir and transported to the laboratory. The carcasses were sent to the Department of Pathological Anatomy and Pathological Physiology, University of Veterinary Medicine and Pharmacy, Košice, Slovak Republic. The overview of the experimental design is shown in Figure 1.

### 2.4 Parasitological techniques

Fecal samples were collected rectally on D14, D20, D28, D42, D56, D70, D84, D98, and D107 *post*-infection. The number of *H. contortus* eggs per gram (EPG) of feces was quantified using a modified McMaster method with a sensitivity of 50 eggs per gram (33). The percent fecal egg count reduction (% FECR) in the CONTROL and ZINC groups was calculated using the formula (34):


%FECR=100×(1−[T2/T1])

where T1 is the mean EPG on D28, and T2 is the mean EPG on the following sampling days (D42, D56, D70, D84, D98, and D107) in treated group, with no control group.

The slaughtered animals were necropsied on D107. The gastrointestinal tract of each lamb was examined to count the total number of adult *H. contortus* in the abomasum. The abomasum of each animal was removed and dissected, and the contents were washed with warm physiological saline and emptied into a jar. The contents were mixed continuously to prevent the clustering of nematodes. Washings were brought up to a volume of 2 L with physiological saline. Three 40-mL aliquots (2%) were collected from each animal and fixed with helminthological iodine. The number of adult *H. contortus* was counted for each animal.

### 2.5 Ruminal and microbial analysis

The fresh ruminal contents were collected at a slaughterhouse and immediately transported to the laboratory in a 39 °C preheated water bath. Ruminal pH was measured using an InoLab pH meter Level 1 (Xylem Analytics, Weilheim, Germany). A liquid sample (4 mL) was collected for the subsequent analysis of ammonia nitrogen. The concentrations of NH_3_N in the samples were determined using the phenol-hypochlorite method (35). The concentrations of short-chain fatty acids (SCFAs) were determined using a PerkinElmer Clarus 500 gas chromatograph (PerkinElmer, Inc., Shelton, USA) as previously described (7). The ruminal content for specific enzymatic activities was immediately frozen at 80 °C after collection, and thawed before homogenization and diluted in an amount of approximately 6 g with 2 mL of a phosphate–citrate buffer solution (pH 6.8) with cOmplete ™ mini EDTA-free Protease Inhibitor Cocktail (Roche Diagnostics GmbH, Mannheim, Germany). The contents were homogenized in an ice bath using a 4,710 series ultrasonic homogenizer (Merck KGaA, Darmstadt, Germany) at a power of 80 W pulsed in one-minute intervals (homogenization and cooling). Specific enzymatic activities for the ruminal microorganisms were determined by the preparation of a cell-free homogenate as previously described (36). Samples for ruminal microbial analyses were isolated using a PureLink Microbiome DNA Purification Kit (Invitrogen, Thermo Fisher, Waltham, USA) according to the manufacturer’s protocol. The concentration of DNA was measured using a NanoDrop spectrophotometer (Thermo Fisher, Waltham, USA). Quantitative PCR was performed on a Roche Light Cycler 480 II (Roche Diagnostics GmbH, Mannheim, Germany) using standard curves for the absolute quantification of specific taxa and total bacteria by amplifying the 16S rRNA gene. The primers used are listed in the Supplementary material. Samples for counting ciliate protozoa were fixed in equal volumes of 8% formaldehyde, and the protozoa were counted and identified microscopically as previously described (37).

### 2.6 Chemical analysis

Standard procedures were used to analyze the dietary substrates in triplicate (38). The dry matter (method no. 930.15) content was obtained by drying the samples at 105 °C for at least 24 h in an oven. The total ash content of the samples was determined by ashing overnight at 550 °C (method no. 942.05) in a muffle furnace. Nitrogen (N) content (method no. 968.06) was determined using a FLASH 4000 Nitrogen/Protein Analyzer (Thermo Fisher Scientific, Cambridge, UK). Crude-protein content was calculated by multiplying the total N content by 6.25 (method no. 990.03). The contents of acidic-detergent fiber and neutral-detergent fiber were analyzed as previously described (39) using an ANKOM 2000 Fiber Analyzer (ANKOM Technology, Macedon, USA) with heat-stable *α*-amylase. The chemical compositions of the meadow hay, barley, and barley with zinc supplements are presented in Table 2.

**Table 2:** Chemical composition of the dietary substrates (*n* = 3).

	Pasture (harvested May–August)		Concentrate	
	CONTROL plot	ZINC plot	–ZnO-NPs	+ZnO-NPs
DM (g/kg)	898	895	881	876
Chemical composition (g/kg DM)				
NDF	414	423	207	222
ADF	317	320	104	81.7
CP	120	127	165	156
N	19.2	19.9	26.4	25.0
Ash	121	128	51.1	52.2

CONTROL plot, a plot consisting exclusively of grassland with grazing lambs fed a diet without ZnO-NPs; ZINC plot, a plot consisting exclusively of grassland, with grazing lambs fed a diet enriched with ZnO-NPs; –ZnO-NPs, concentrate without zinc nanoparticles; +ZnO-NPs, concentrate with zinc nanoparticles; DM, dry matter; NDF, neutral-detergent fiber; ADF, acid-detergent fiber; CP, crude protein; N, nitrogen.

### 2.7 Histology

Histological examinations were performed similarly to those previously described (40). Samples of fresh ruminal tissues (approximately 3 × 3 cm) were washed in a phosphate buffer (0.1 M, pH 7.4), put in plastic containers, and fixed in a 10% buffered formalin solution as pieces of tissue spread on flat polystyrene. The fixed material was processed using a series of reagents and embedded in Paraplast PLUS paraffin blocks (Leica, Buffalo Grove, USA), which were then cut with a rotary microtome into sections 3.5 μm thick. Slides with a paraffin section were automatically stained with hematoxylin and eosin (Varistain Gemini, Thermo Scientific, Runcorn, UK). An Axio Lab. A1 microscope (Carl Zeiss, Jena, Germany) equipped with a Zeiss Axiocam ERc5s digital camera was used for histological evaluation. Photographs were analyzed and recorded using ZEN 2.3 (blue edition) software (Carl Zeiss Microscopy GmbH, Jena, Germany).

### 2.8 Statistical analysis

Data were statistically analyzed using an unpaired *t*-test (GraphPad Prism 9.2.0 (332) 2021; GraphPad Software, Inc., San Diego, USA). Results were considered significant at *p <* 0.05.

## 3 Results

### 3.1 Vegetation records

The phytosociological relevés, the number of species, and the percentages of coverage represented by species with defined characteristics are presented in Table 1. The plant taxa identified in pastures and the coverage of individual taxa on the pastures were used as descriptors to investigate the differentiation of the vegetation based on medicinal properties and nutritional values. The number of species used in homeopathy and folk medicine was in both pastures from 14 to 15 species. The CONTROL pasture contained remarkably higher (11 plant species listed in the pharmacopeia) than other variants (5 and 4 species, respectively). Plants used as recognized and standardized medicines were present in small numbers, 2 or 3 species in pastures, but covered larger areas in the ZINC pasture (5 − 12.5%) compared to the CONTROL pasture. Unsuitable, harmful, and very harmful plants were rare (coverage up to 1%).

### 3.2 Principal component analysis

**Figure 2:**
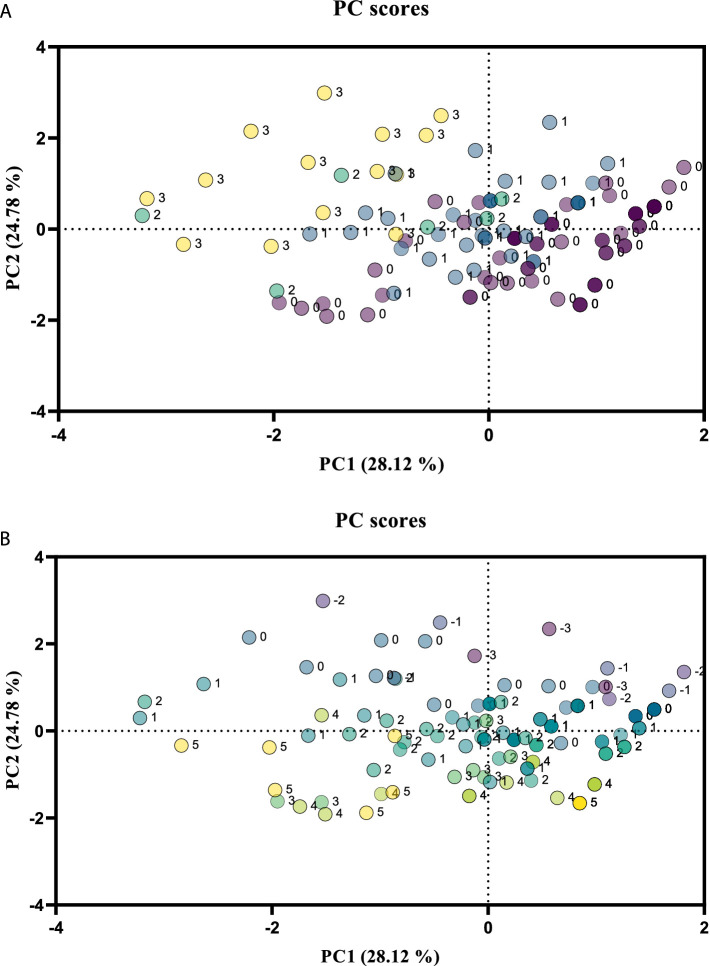
Plot of the scores for the first two principal components (PCs) for the medicinal properties and nutritional values of the plant species analyzed based on vegetation relevés in pastures of the CONTROL and ZINC groups of lambs. Scale of the medicinal properties **(A)**: 1, used in homeopathy and folk medicine; 2, recognized and regulated drugs; 3, official according to the pharmacopeia. 0, plants without medicinal properties that have no documented therapeutic, aromatic, or culinary uses for health purposes. Scale of the nutritional value **(B)**: 1, low; 2, low to medium; 3, good; 4, outstanding; 5, excellent; −1, unsuitable; −2, harmful; −3, dangerous.

A data matrix was created for the PCA, with rows representing the 117 plant taxa analyzed, and columns representing individual relevés with the coverage of individual taxa on the pastures, medicinal properties, and nutritional values as selected components. The first two principal components (PCs) were extracted, with PC1 and PC2 explaining 28.12 and 24.78% of the total variance, respectively. The corresponding scores obtained from an eigenanalysis of the covariance matrix of the automatically scaled data were plotted. PC1 and PC2 effectively separated the plant species with medicinal properties (Figure 2A) and nutritional values (Figure 2B). The majority of the plants with medicinal properties had positive PC2 scores. Most plants with outstanding nutritional values had negative PC1 scores. A detailed examination of the variable loadings of PC1 indicated that plant coverage in the ZINC pasture and the nutritional values of the plants contributed the most to PC1, and that plant coverage in the CONTROL pasture and the medicinal properties of the plants contributed the most to PC2.

### 3.3 Parasitological status

**Figure 3:**
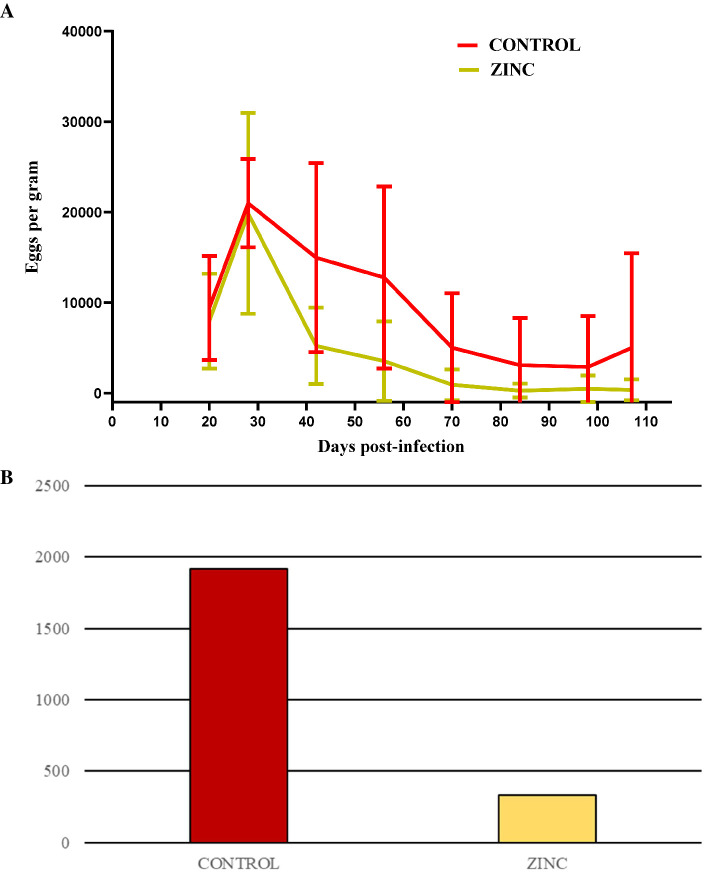
**(A)** Mean fecal egg counts ± SD for the CONTROL and ZINC groups of lambs infected with *Haemonchus contortus.* **(B)** Abomasal worm counts of *H. contortus* in the lambs in both groups at the end of the experiment (D107) (*p >* 0.05).

All lambs had similar initial total body weights (BWs) (Table 3). Weight parameters (BWs, live-weight gains (LWGs), and daily weight gains (DWGs)) did not differ significantly between groups; however, DWG on D107 in the ZINC group shows an upward trend. The average egg count in lamb groups peaked on D28 *post*-infection, and egg excretion gradually decreased from D28 onward, indicating parasite elimination. There was a significant decrease in egg shedding from D42 onwards in the ZINC group, and from D56 and D70 onwards in the CONTROL group. Egg shedding in the ZINC group at the end of the experiment had decreased by up to 98%. In contrast, egg shedding in the CONTROL group at D107 had decreased by only 67% (Figure 3A and Table 4). The abomasal worm counts in the lambs were lower in the ZINC group but did not differ significantly from the counts in the CONTROL group (*p >* 0.05) (Figure 3B).

**Table 3:** Effect of pastures and diets on the growth parameters in the control (CONTROL) and ZnO-NPs (ZINC) groups of lambs (mean ± SD, *n* = 10).

	Day	CONTROL	ZINC	*p*
BW (kg)	D0	15.9 ± 2.65	16.4 ± 2.92	0.693
	D28	20.2 ± 4.17	20.9 ± 4.05	0.708
	D56	24.4 ± 4.95	24.6 ± 4.41	0.927
	D84	27.6 ± 5.83	27.4 ± 4.59	0.933
	D107	30.2 ± 6.60	30.6 ± 5.48	0.884
LWG (kg)	D28	4.34 ± 1.957	4.48 ± 1.424	0.857
	D56	4.15 ± 1.164	3.71 ± 1.502	0.473
	D84	3.23 ± 1.440	2.76 ± 1.129	0.427
	D107	2.57 ± 1.288	3.23 ± 1.352	0.278
DWG (g/d)	D28	155 ± 69.9	160 ± 50.9	0.812
	D56	143 ± 40.1	128 ± 51.8	0.437
	D84	120 ± 53.3	102 ± 41.8	0.338
	D107	112 ± 56.0	140 ± 58.8	0.074

SD, standard deviation; BW, body weight; LWG, live-weight gain; DWG, daily weight gain.

**Table 4:** Percent fecal egg count reduction (%FECR) in the control (CONTROL) and ZnO-NPs (ZINC) groups of lambs (*n* = 10).

	**%FECR in days (D) *post*-infection**					
	**D42**	**D56**	**D70**	**D84**	**D98**	**D107**
CONTROL	28.61	39.14	76.08	85.25	86.23	76.16
*p*	0.117	0.032	<0.001	<0.001	<0.001	0.004
ZINC	73.74	82.19	95.39	98.64	97.58	98.13
*p*	0.011	0.004	<0.001	<0.001	<0.001	<0.001

%FECR, expressed as a percentage of egg reduction to day 28 post-infection.

### 3.4 Ruminal fermentation and microbiota

The ruminal concentration of NH_3_N (*p* = 0.018), *n-*butyrate (*p* = 0.025), *n-*valerate (*p* = 0.002), total protozoa count (*p <* 0.001), and the enzymatic activities of *α*-amylase (*p <* 0.001) and xylanase (*p* = 0.006) were significantly higher in the ZINC group than in the CONTROL group (Table 5). The molar proportion of acetate was significantly lower (*p* = 0.011) in the ZINC group than in the CONTROL group. The relative abundances of *Butyrivibrio proteoclasticus, Ruminococcus albus, Fibrobacter succinogenes, R. flavefaciens, B. fibrisolvens*, *Prevotella*, *Streptococcus bovis,* and total *Methanogens* did not differ significantly (*p >* 0.05) among the groups.

**Table 5:** Effect of pastures and diets on parameters of ruminal fermentation in the control (CONTROL) and ZnO-NPs (ZINC) groups of lambs (mean ± SD, *n* = 10).

	CONTROL	ZINC	*p*
pH	6.61 ± 0.300	6.70 ± 0.133	0.393
NH_3_N (mg/L)	149 ± 51.6	180 ± 34.9	0.018
Total SCFA (mM/L)	36.6 ± 5.32	36.0 ± 13.29	0.249
Acetate (molar%)	64.7 ± 1.84	62.0 ± 2.45	0.011
Propionate (molar%)	18.2 ± 1.08	19.7 ± 1.23	0.389
*iso*-Butyrate (molar%)	1.68 ± 0.184	1.56 ± 0.364	0.384
*n*-Butyrate (molar%)	12.8 ± 1.41	13.2 ± 1.21	0.025
*iso*-Valerate (molar%)	1.63 ± 0.268	1.92 ± 0.481	0.113
*n*-Valerate (molar%)	1.95 ± 0.267	2.65 ± 0.563	0.002
A: P ratio	3.78 ± 0.290	3.54 ± 0.378	0.123
Total protozoa (10^5^/g WRC)	52.3 ± 23.10	81.1 ± 15.46	*<* 0.001
Specific enzymatic activities of the ruminal microorganisms (μkat/g protein)			
α-Amylase	2.09 ± 1.302	3.68 ± 0.913	*<* 0.001
Carboxymethyl-cellulase	0.412 ± 0.180	0.418 ± 0.202	0.940
Xylanase	36.5 ± 25.01	59.6 ± 25.07	0.006

SD, standard deviation; NH_3_N, ammonia nitrogen; SCFA, short-chain fatty acids; wRC, count per gram wet ruminal content.

### 3.5 Histology

**Figure 4:**
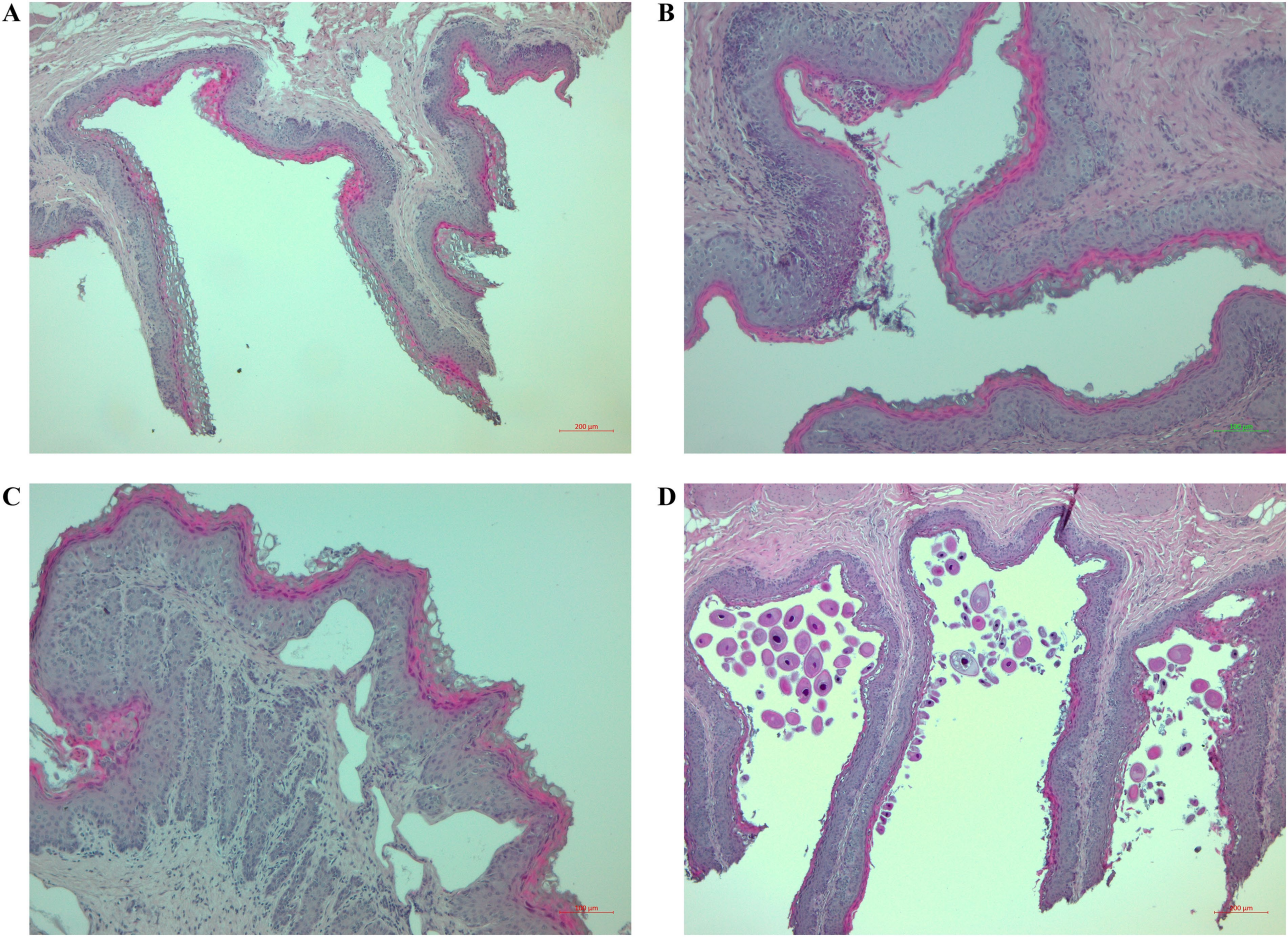
Histological changes to the ruminal tissues of the lambs. **(A)** Sections stained with hematoxylin and eosin (H&E) (40×) showing papillary degradation, rough keratinocytic layers, and infiltration of lymphocytes in the epithelium and lamina propria. **(B)** Sections stained with H&E (100×) showing lymphocytic and eosinophilic infiltration of ruminal papillae with epithelial damage. **(C)** Sections stained with H&E (100×) showing emphysema in the lamina propria. **(D)** Sections stained with H&E (40×) showing numerous protozoa and degradation of papillae.

The size of the ruminal papillae varied in all lambs. The lambs in the CONTROL group mostly had thin and medium-length papillae, and the lambs in the ZINC group had papillae of different lengths and sizes. The homogeneity of the papillae was affected in both groups, but papillary degradation (various degrees) was present only occasionally (Figure 4A). The epithelial keratinocytic layer was thin and flat in the CONTROL group, with sections of balloon-shaped keratinocytes. The epithelial keratinocytic layer in the ZINC group was mostly rough. Inflammation of the lamina propria with infiltrates of inflammatory cells, mainly lymphocytes, was present in both groups, but the epithelium was inflamed with lymphocytes and eosinophils only in the ZINC group (Figure 4B). Only one lamb in the CONTROL group had connective-tissue edema and lamina propria emphysema (Figure 4C). Organisms with morphologies similar to *Balantidium coli* were present in both groups (Figure 4D).

## 4 Discussion

### 4.1 Vegetation

In the vegetation of pastures highly dominate native species, either those occurring only in natural or semi-natural communities (proanthropophytes; e.g., *Galium mollugo*, *Polygala vulgaris*, *Potentilla erecta*) or native species that could also grow in secondary, synanthrop or ruderal stands (apophytes, e.g., *Achillea millefolium*, *Agrimonia eupatoria*, *Lysimachia nummularia*, *Plantago lanceolata*, and *Trifolium repens*). In the studied plots, we recorded species-rich plant communities with species richness ranging from 49 to 59 species of vascular plants in an area of 25 m^2^. A quite high number of these species are used in human medicine, with 20–30 species per plot classified in one of three categories of medicinal plants. This gives good potential for medicinal effects, also for animals such as the lambs used in our experiment, and thus for supporting their recovery after being infected with parasites. Our results suggest that the species composition of plants in the pastures of the Control and Zinc groups of lambs is partially different, which may bias our results. However, the results in Table 2 demonstrate that the chemical/nutritional composition of the pastures is very similar overall. This suggests that the more pronounced anthelmintic effects observed were probably due to the ZnO-NPs treatment. In order to draw more specific conclusions, a deeper analysis is needed, including the study of chemical compounds that may play an active role in promoting the recovery of the studied animals.

The PCA allowed us to distinguish plants used in homeopathy and folk medicine (e.g., *Centaurea jacea*, *Knautia arvensis*, and *Veronica chamaedrys*), plants recognized and regulated as medicinal products (e.g., *Betonica officinalis*, *Prunella vulgaris*, and *Trifolium repens*), and plants officially used according to the pharmacopeia (e.g., *Fragaria viridis*, *Plantago lanceolata*, and *Potentilla erecta*) in pastures (Figure 2A). Communities dominated by plants with medicinal properties were mainly in the CONTROL pasture, where the vegetation was rich in bioactive compounds with direct and indirect anthelmintic effects (12). Communities dominated by plants with nutritional values were mainly in the ZINC pasture. The PCA also separated plants with low nutritional value (e.g., *Betonica officinalis*, *Luzula campestris*, and *Thymus pulegioides*), low-to-medium nutritional value (e.g., *Anthoxanthum odoratum*, *Campanula patula*, and *Holcus lanatus*), those with good (*Agrostis capillaris*, *Hieracium pilosella*, and *Vicia cracca*) or very good (*Arrhenatherum elatius*, *Pimpinella saxifraga*, and *Trifolium medium*) nutritional value, and those with excellent nutritional value (e.g., *Plantago media*, *Trifolium repens*, and *Festuca pratensis*) (Figure 2B). Unsuitable (*Ranunculus polyanthemos*), harmful (*Hypericum perforatum*), or very harmful (*Euphorbia cyparissias*) plants occurred only sporadically.

### 4.2 Effect on parasitological status

The present experiment did not confirm the effect of zinc nanoparticle supplementation on weight parameters in infected lambs, but DWG on D107 in the ZINC group suggests a potential growth increase. Mena et al. (41) found that a daily supply of 150 mg of ZnO-NPs favored weight gain in young sheep naturally infected with GINs.

The parasitological benefits of grazing on grassland can persist in the long term, potentially providing a sustainable method of controlling gastrointestinal nematodes. The enrichment of grassland, e.g., with sown chicory, provides high-quality forage that is rich in some trace elements (12, 42). Flavonoids affect zinc metabolism by forming zinc complexes that facilitate zinc transport, potentially leading to biological consequences and health benefits (43). The mechanism of action of ZnO-NPs involves influencing the antioxidant system of *H. contortus* by inducing severe oxidative stress, resulting in the denaturation of antioxidant enzymes (26). Building on our previously published findings, our results underscore the dependence of the grazing effect on the diversity and synergy of plant polyphenols. Combining polyphenols with nanozinc may lead to enhanced therapeutic effects, as suggested by the reduced egg production in the ZINC groups, where these values already decreased significantly from D42.

In our previous experiment (12), we found that a reduction in the level of pasture contamination with *H. contortus* third-stage larvae could be associated with the influence of bioactive plants. EPG levels in our experiment peaked at D28 (19,880 − 20,990) for both groups, and high levels of egg shedding in lambs were maintained for at least 1 week (D20–D28), implying relatively high pasture contamination, but the animals in the ZINC group were not re-infected. Egg excretion in all animals in the ZINC group was radically or gradually reduced. In contrast, a few animals in the CONTROL group maintained higher EPG levels up to D107. This finding suggests that zinc supplementation may also contribute to increased protective function in animals before re-infection on pasture.

The control of helminth infections in grazing ruminant animals in the future will be different compared with now, because we will potentially rely less on anthelmintics due to the availability of different control tools, such as bioactive nutraceuticals. Our results suggest that bioactive compounds in semi-natural grassland may have a high potential to reduce parasitic burdens. If this approach is further potentiated by supplementation with trace elements such as zinc, parasitic burdens could be almost eliminated in a relatively short time.

### 4.3 Effect on ruminal fermentation and microbiota

Ruminal fermentation in lambs in our study changed in response to endoparasitic infection. Ruminal ammonia nitrogen is normally the most abundant nitrogenous compound needed for microbial growth, and its increased values in the rumen of the ZINC group of lambs may have been due to the lower consumption of ammonia nitrogen by microorganisms. These microorganisms have access to a readily available source of energy, which increases microbial protein synthesis or decreases the use of amino acids as a microbial energy source (44). A large increase in the amount of additional endogenous nitrogen entering the duodenum in lambs infected with *H. contortus* would probably lead to the loss of amino acids because the reabsorbed N not from ammonia would likely be used inefficiently (45). In this experiment, the optimal level of ammonia-N in the rumen, i.e., 60–290 mg/L (46), was not exceeded, which can vary and depends on the optimal concentration of ammonia nitrogen for maximum fermentation rate and maximum microbial protein production (47). Animals infected with *H. contortus* either have lower ruminal concentrations of propionate, as in our study, or experience a shift in the production of SCFAs toward propionate in response to a mixed infection with *H. contortus* and *Trichostrongylus colubriformis* (48). Supplementation with ZnO-NPs in the ZINC group, however, shifted the pattern of fermentation toward a decrease in the molar proportion of acetate and an increase in the molar proportion of *n*-valerate and *n-*butyrate compared to the CONTROL group. ZnO-NP supplementation can alter the ruminal fermentation process and increase feed energy by producing SCFAs (49), but the acetate: propionate ratio probably has little impact on the energy efficiency of lamb production (50). The addition of zinc to the diets of ruminants can strongly affect ruminal fermentation and microbiota, depending on the dosage, form, and duration of supplementation (21, 40, 51). The lack of significant differences in ruminal microbial population in the present experiment was consistent with our previous results with ZnO-NPs in uninfected lambs (21). The increased number of ruminal protozoa in the ZINC group probably assimilated ZnO-NPs preferentially, potentially shifting ruminal fermentation toward protozoa at the expense of bacteria (52, 53). ZnO, however, is poorly assimilated by ruminal bacteria and protozoa, which preferentially assimilate highly soluble zinc (54). The administration of ZnO in the form of nanoparticles nevertheless enabled lambs to rapidly adapt to a zinc-rich diet (21, 46), and the relative abundance of bacteria in the ZINC group remained unaffected. The currently recommended levels of zinc intake, however, are defined to meet the needs of the animal, rather than those of the ruminal microbiota (10). The latter will likely require little Zn supplementation (54). The enzymatic activities of *α*-amylase and xylanase increased in the ZINC group. Ruminal microbiota, especially the more abundant protozoa in the ZINC group, were probably more associated with increased α-amylase and xylanase activities, which would accelerate biodegradation during substrate processing in the rumen (55). Protein metabolism in animals infected with endoparasites, however, is more affected by GIN infection than is fiber metabolism (56), so GIN infection will likely increase ruminal carbohydrate metabolism. This increase may have compensated for the loss of energy supply caused by reduced protein and lipid metabolism due to infection (57). Finally, zinc supplementation also promotes the efficient ruminal digestion of complex substrates, which requires the coordinated action of many enzymes that can act individually and synergistically, or individual enzymes can assemble into multienzyme complexes (58). Additionally, supplements with nano-zinc oxide lead to faster and more effective regeneration than regular zinc oxide and have a stronger effect on immunity and bacteria (59, 60).

### 4.4 Effect on ruminal histology

The growth and development of the ruminal papillae in the present experiment depended on the type of diet in lambs. SCFAs are absorbed through the ruminal epithelium, which is responsible for absorbing and metabolizing nutrients and microbial by-products, so the rate of absorption is influenced by the concentration and surface area of the papillae and by the availability of transport proteins (61, 62). Active fermentation and SCFAs generally induce morphofunctional changes in ruminal papillae, stimulate their development (63, 64), and can probably also affect the keratinocytic epithelial layer and connective-tissue edema, especially in lambs supplemented with ZnO-NPs (21). The morphology of ruminal papillae is not associated with average daily gain or feed intake, but evidence suggests that a more active gastrointestinal tract is linked to genes associated with higher feed efficiency. This evidence supports the idea of a relationship between metabolic activity and the function and structure of the ruminal epithelium (65). Lambs in the CONTROL group had a thin, flat keratinocytic epithelial layer with balloon-shaped keratinocytes, indicating lower metabolic and functional activities in the papillary epithelium (66). In contrast, the keratinocytic epithelial layers of the lambs in the ZINC group were predominantly rough. Inflammation of the lamina propria with infiltrates of inflammatory cells, primarily lymphocytes, however, was evident in both groups. The ZINC group mainly exhibited epithelial inflammation with lymphocytes and eosinophils. These results are consistent with our previous findings in uninfected lambs supplemented with ZnO-NPs (21). We previously reported adverse effects on the health of the ruminal epithelium after 70 d of zinc (organic zinc form) supplementation combined with a mixture of medicinal plants (40). Inflammation of the lamina propria and degradation of the papillae leads to infiltration by leukocytes and eosinophils and secondary infection of the rumen by the resident microbial population (67), in some cases consistent with our results. In addition, organisms with *B. coli*-like morphology were present in both groups of lambs, which is consistent with our previous histopathological observations in the rumen of GIN-infected lambs (68). The ability of host ruminal epithelial cells to transport SCFAs nevertheless remained unaffected. Finally, *H. contortus* infections generally damage the abomasal mucosa, but damage to ruminal tissue is not relevant in terms of clinical relevance in infected lambs.

## 5 Conclusion

Phytosociological relevés identified the medicinal properties and nutritional value of plants in semi-natural grasslands. Active ruminal fermentation in the ZnO group had a slightly negative impact on the health of the ruminal papillae and epithelium. However, the dynamics of *H. contortus* infections were reduced due to the medicinal properties and nutritional value of the pasture plants, as well as ZnO nanoparticle supplementation, which exhibited strong anthelmintic properties. Although ZnO nanoparticles show promise as anthelmintics, their toxicity to organisms and the environment must also be considered. In conclusion, the initial recommendation for small ruminant farmers should be to graze their herds on pastures rich in medicinal plants (if possible). Nevertheless, even short-term administration of microelements such as zinc can support the anthelmintic effect synergistically and protect animals from parasitic infection while grazing.

## Data Availability

The original contributions presented in the study are included in the article/Supplementary material; further inquiries can be directed to the corresponding authors.
