# Supplementary material for: Effect of zinc oxide nanoparticle supplementation on parasite infection and rumen environment of grazing lambs
Source: Front Vet Sci. 2025 Oct 30;12:1684585. doi: 10.3389/fvets.2025.1684585 (PMC12611691; doi:10.3389/fvets.2025.1684585)
Supplement: Supplementary file 4 [file Data_Sheet_1.pdf]

Supplementary Material

Table: The sequences of primers specific to the analyzed bacteria species

Species	Primer sequences	Reference
Ruminococcus flavefaciens	F – 5' CGAACGGAGATAATTTGAGTTTACTTAGG 3'<br>R – 5' CGGTCTCTGTATGTTATGAGGTATTACC 3'	[1]
Fibrobacter succinogenes	F – 5' GTTCGGAATTACTGGGCGTAAA 3'<br>R –5' CGCCTGCCCCTGAACTATC 3'	[2]
Streptococcus bovis	F – 5' TTCCTAGAGATAGGAAGTTTCTTCGG 3'<br>R – 5' ATGATGGCAACTAACAATAGGGGT 3'	[3]
Butyrivibrio proteoclasticus	F – 5' TCCTAGTGTAGCGGTGAAATG 3'<br>R –5' TTAGCGACGGCACTGAATGCCTA 3'	[4]
Ruminococcus albus	F – 5' CCCTAAAAGCAGTCTTAGTTCG 3'<br>R – 5' CCTCCTTGCGGTTAGAACA 3'	[5]
Butyrivibrio fibrisolvens	F – 5' ACACACCGCCCGTCACA 3'<br>R – 5' TCCTTACGGTTGGGTCACAGA 3'	[6]
Prevotella spp.	F – 5' GAAGGTCCCCCACATTG 3'<br>R – 5' CAATCGGAGTTCTTCGTG 3'	[3]
Total methanogens	F – 5' GAGGAAGGAGTGGACGACGGTA 3'<br>R – 5' ACGGGCGGTGTGTGCAAG 3'	[7]
16 S V4	F – 5' TATGGTAATTGTGTGNCAGCMGCCGCGGTAA 3'<br>R – 5' AGTCAGTCAGCCGGACTACHVGGGTWTCTAAT 3'	[8]

Supplementary Material

1. Zeng J, Bian Y, Xing P, Wu QL. Macrophyte species drive the variation of bacterioplankton community composition in a shallow freshwater lake. *Appl Environ Microbiol*. (2012) 78:177–184. https://doi.org/10.1128/AEM.05117-11
2. Denman SE, McSweeney CS. Development of a real-time PCR assay for monitoring anaerobic fungal and cellulolytic bacterial populations within the rumen. *FEMS Microbiol Ecol.* (2006) 58:572–582. https://doi.org/10.1111/j.1574-6941.2006.00190.x
3. Yu Y, Lee C, Kim J, Hwang S. Group-specific primer and probe sets to detect methanogenic communities using quantitative real-time polymerase chain reaction. *Biotechnol Bioeng.* (2005) 89:670–679. https://doi.org/10.1002/bit.20347
4. Potu RB, AbuGhazaleh AA, Hastings D, Jones K, Ibrahim SA. The effect of lipid supplements on ruminal bacteria in continuous culture fermenters varies with the fatty acid composition. *J Microbiol.* (2011) 49:216–223. https://doi.org/10.1007/s12275-011-0365-1
5. Wang RF, Cao WW, Cerniglia CE. PCR detection of *Ruminococcus* spp. in human and animal faecal samples. *Mol Cell Probes*. (1997) 11:259–265. https://doi.org/10.1006/mcpr.1997.0111
6. Li M, Penner GB, Hernandez-Sanabria E, Oba M, Guan LL. Effects of sampling location and time, and host animal on assessment of bacterial diversity and fermentation parameters in the bovine rumen. *J Appl Microbiol.* (2009) 107:1924–1934. https://doi.org/10.1111/j.1365-2672.2009.04376.x.
7. Koike S, Ueno M, Miura H, Saegusa A, Inouchi K, Inabu Y, Sugino T, Guan LL, Oba M, Kobayashi Y. Rumen microbiota and its relation to fermentation in lactose-fed calves. J Dairy Sci. (2021) 104:10744-10752. https://doi.org/10.3168/jds.2021-20225
8. Poeker SA, Geirnaert A, Berchtold L, Greppi A, Krych L, Steinert RE, de Wouters T, Lacroix C. Understanding the prebiotic potential of different dietary fibers using an *in vitro* continuous adult fermentation model (PolyFermS). *Sci Rep.* (2018) 8(1):4318. https://doi:10.1038/s41598-018-22438-y
